# Transcriptome and metabolome analysis to reveal major genes of saikosaponin biosynthesis in *Bupleurum chinense*

**DOI:** 10.1186/s12864-021-08144-6

**Published:** 2021-11-19

**Authors:** Yilian He, Hua Chen, Jun Zhao, Yuxia Yang, Bin Yang, Liang Feng, Yiguan Zhang, Ping Wei, Dabin Hou, Junning Zhao, Ma Yu

**Affiliations:** 1 School of life science and engineering, Southwest University of Science and Technology, 59 Qinglong Road, Mianyang, 621010 Sichuan China; 2 Institute of Medicinal Plant Resources, Sichuan Academy of Traditional Chinese Medicine Sciences, 51 4th Section S. Renmin Road, Chengdu, 610041 Sichuan China; 3 Sichuan Institute for Translational Chinese Medicine, Chengdu, 610041 China; 4 Laboratory of Medicinal Plant Cultivation, Institute of Medicinal Plant Development (IMPLAD), Chinese Academy of Medical Sciences & Peking Union Medical College, Beijing, 100193 China

**Keywords:** *Bupleurum chinense* DC. Transcriptome, Metabolome, Saikosaponin biosynthetic pathway

## Abstract

**Background:**

*Bupleurum chinense* DC. is a widely used traditional Chinese medicinal plant. Saikosaponins are the major bioactive constituents of *B. chinense*, but relatively little is known about saikosaponin biosynthesis. In the present study, we performed an integrated analysis of metabolic composition and the expressed genes involved in saikosaponin biosynthetic pathways among four organs (the root, flower, stem, and leaf) of *B. chinense* to discover the genes related to the saikosaponin biosynthetic pathway.

**Results:**

Transcript and metabolite profiles were generated through high-throughput RNA-sequencing (RNA-seq) data analysis and liquid chromatography tandem mass spectrometry, respectively. Evaluation of saikosaponin contents and transcriptional changes showed 152 strong correlations (*P* < 0.05) over 3 compounds and 77 unigenes. These unigenes belonged to eight gene families: the acetoacetyl CoA transferase (AACT) (6), HMG-CoA synthase (HMGS) (2), HMG-CoA reductase (HMGR) (2), mevalonate diphosphate decarboxylase (MVD) (1), 1-deoxy-D-xylulose-5-phosphate synthase (DXS) (3), farnesyl diphosphate synthase (FPPS) (11), β-amyrin synthase (β-AS) (13) and cytochrome P450 enzymes (P450s) (39) families.

**Conclusions:**

Our results investigated the diversity of the saikosaponin triterpene biosynthetic pathway in the roots, stems, leaves and flowers of *B. chinese* by integrated transcriptomic and metabolomic analysis, implying that manipulation of P450s genes such as *Bc95697* and *Bc35434* might improve saikosaponin biosynthesis. This is a good candidate for the genetic improvement of this important medicinal plant.

**Supplementary Information:**

The online version contains supplementary material available at 10.1186/s12864-021-08144-6.

## Background

Plants of the genus *Bupleurum* are major components of Oriental folk medicine in China, Japan and Korea, and have been officially listed in the Chinese and Japanese Pharmacopoeias [1]. These species are used either alone or in combination with other ingredients for treatment of the common cold, inflammatory disorders, hepatitis and fever [2]. Up to now, phytochemical studies have shown that *Bupleurum* contains abundant natural compounds, such as phenolics, lignans, terpenoids (triterpenoids and sterols), mono- and sesquiterpenes (essential oils) and polyacetylenes [3]. Among them, saikosaponins (SSs) are considered as the principal bioactive components [4]. SSs generally constitute the main class of secondary metabolites in the genus *Bupleurum*, accounting for up to 7% of the total dry weight of the roots [2].

**Fig. 1:**
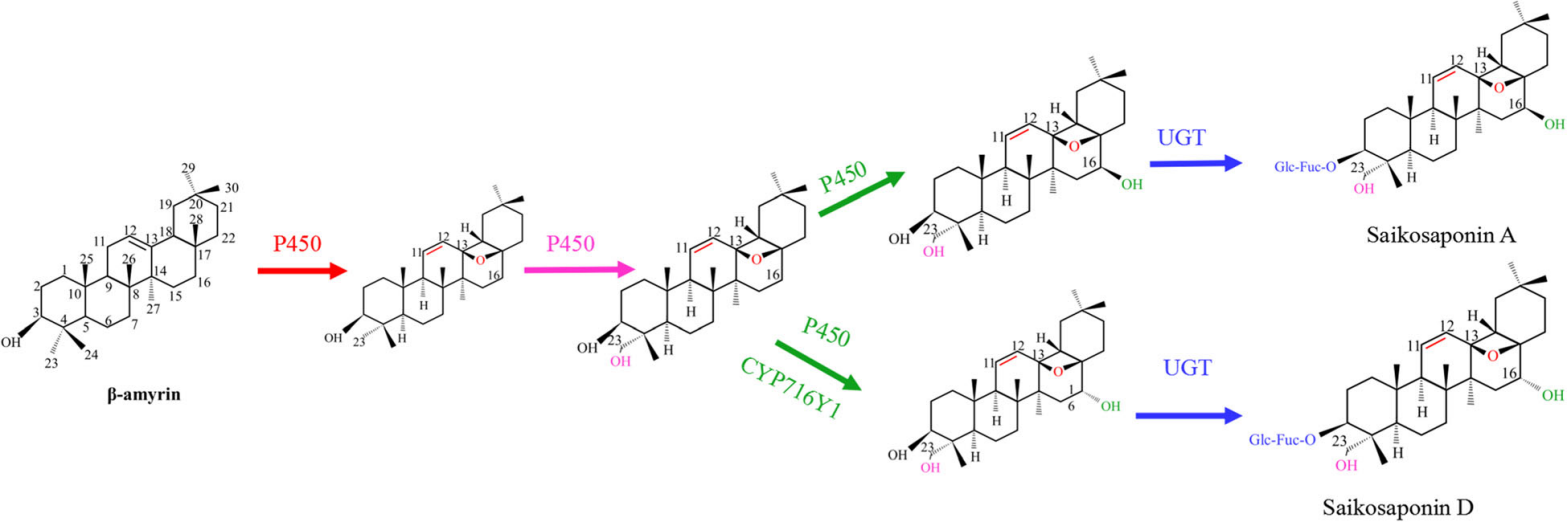
Diagrammatic representation of the biosynthetic pathway for triterpenoid saponins in *B. chinense*. The universal precursor β-amyrin is converted by a series ofoxidative reactions to saikosaponin A and saikosaponin D. P450, cytochromes P450; UGT, uridine diphosphate glycosyltransferases

In recent years, more than 120 glycosylated oleanane-type and ursane-type SSs have been isolated from *Bupleurum* species [5]. A representative biosynthetic pathway for triterpenoid saponins in *Bupleurum* was described by Zheng et al., [6]: isopentenyl diphosphate (IPP) and dimethylallyl diphosphate (DMAPP) are produced by the mevalonate (MVA) pathway and the methylerythritol phosphate (MEP) pathway. IPP and DMAPP synthesize 2,3-oxidosqualene, which is catalyzed by geranyl pyrophosphate synthetase (GPS), FPPS, squalene synthetase (SS) and squalene epoxidase (SE). 2,3-Oxidosqualene is cyclized by β-AS to produce β-amyrin. Finally, β-amyrin is oxidatively modified and glycosylated to produce various types of SSs by P450s and uridine diphosphate glycosyltransferases (UGTs) (Fig. 1). In our previous studies, candidate genes responsible for saikosaponin biosynthesis were identified in *B. chinense* on the basis of the coordinated upregulation of their expression with β-AS in methyl jasmonate-treated adventitious roots, especially *P450*s and *UGT*s [7]. However, the regulatory mechanism for the saikosaponin biosynthesis pathway is still unknown.

Integrated omics analysis of the transcriptome and metabolome has become an effective tool to better understand biosynthetic pathways [8]. This technique enables identification of novel functional genes and characterization of the regulatory mechanisms and key factors of biosynthetic pathways. The regulatory genes in the flavanol [9], anthocyanin [10] and ginsenoside biosynthesis pathways [11] have been explored using integrated analysis, and potential target genes have been identified.

In this study, we used *B. chinense* to 1) explore the regulatory networks of the saikosaponin synthesis pathway in this species at the level of transcriptome and metabolome, 2) identify the differential expression of saikosaponin metabolites and their regulatory genes among the roots, stems, leaves and flowers at the adult stage, and 3) distinguish the key factors associated with saikosaponin biosynthesis.

## Results

### Metabolic profiling among the root, stem, leaf and flower

**Fig. 2:**
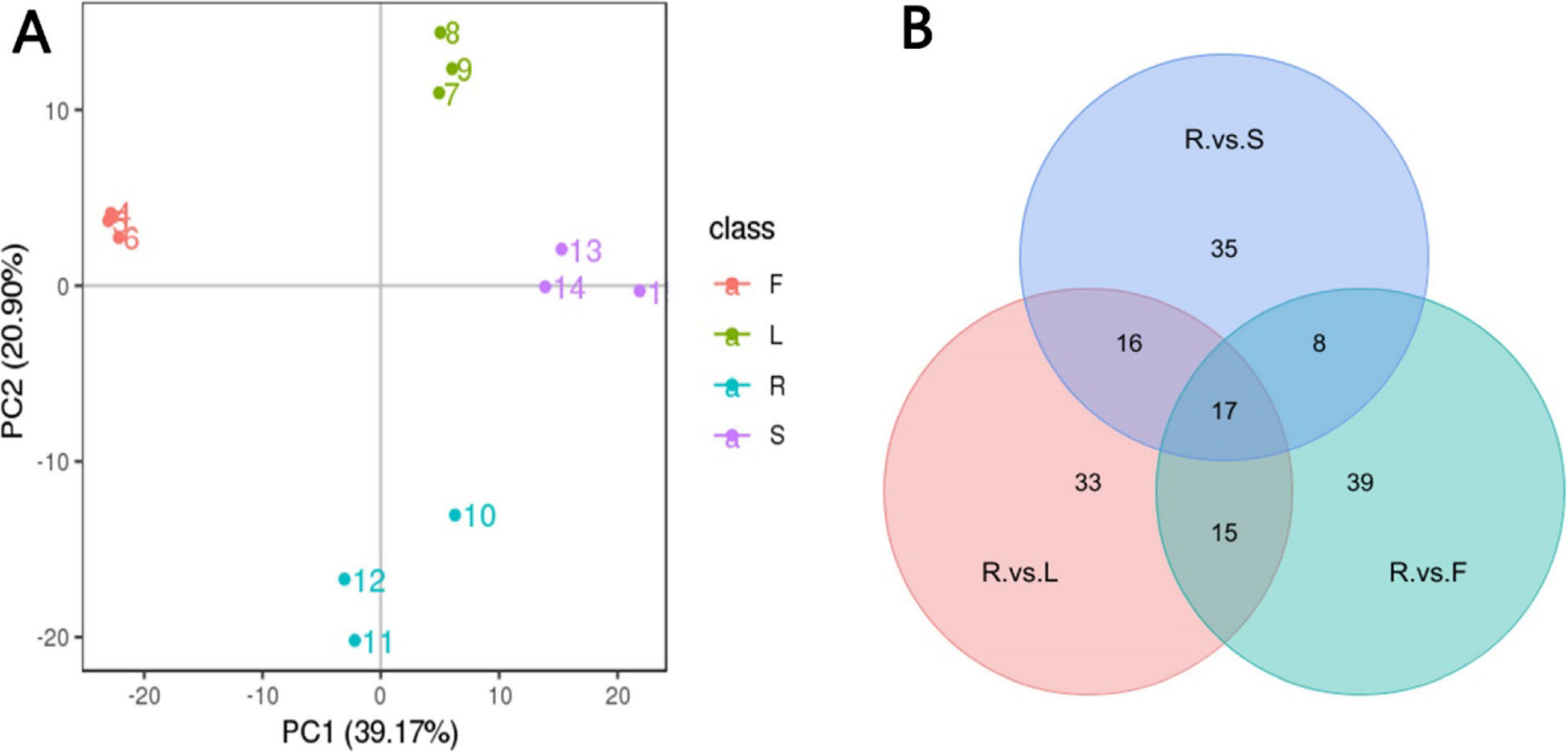
PCA score plot of organs and numbers of potential markers for each. PCA score plots were derived from metabolite ions acquired from ESI+ and ESI- modes (**A**). Potential markers were selected by comparing quantitative differences of mass ions in ESI+ and ESI- modes (**B**) between root and stem, between root and flower, and between root and leaf. F, flower; L, leaf; R, root; and S, stem

A total of 600 metabolites were identified in the samples, and all of them accumulated differentially in the four organs (Additional file 1: Table S1). To compare the compositions of metabolites involved in saikosaponin synthesis in the four organs, datasets obtained from high-performance liquid chromatography-tandem mass spectrometry (HPLC-MS/MS) in the ESI+ and ESI– modes were subjected to Principal component analysis (PCA). The results showed that the four organs were separated in the PC1 × PC2 score plots. Indeed, PC1 and PC2 in ESI+ and ESI– modes (39.17 and 20.90%, respectively) were clearly separated among the roots, stems, leaves and flowers (Fig. 2A). This grouping indicated that the accumulation pattern of metabolites varied among organs. Selection of the variables responsible for the differences was performed through statistical analysis as described in the Materials and Methods. A total of 76, 81, and 79 mass ions were selected between roots and stems, between roots and leaves, and between roots and flowers in the ESI+ and ESI^−^ modes, respectively (Fig. 2B).

We detected four triterpenoids among 10 terpenoids, including saikosaponin A (SSa), saikosaponin B (SSb), saikosaponin D (SSd) and momordin Ic. In addition, two sesquiterpene lactones (artemisinin and atractylenolide I), two monoterpenoids (rehmannioside A and albiflorin), one diterpenoid (kaurenoic acid) and one triterpenic acid (betulonic acid) were detected. The relative abundance of triterpenoids in organs was much higher than other terpenoids, except in the stem. The relative abundance of sesquiterpene lactones in the stem was higher than that in other three organs. SSb and SSd were the most abundant components of the root extracts (Table 1). The root was the most active organ regarding to SSb and SSd accumulation, while flower was the most active organ for SSa.

**Table 1:** Summary of Saikosaponin A (SSa), Saikosaponin B (SSb) and Saikosaponin D (SSd) in root, stem, leaf and flower. F, flower; L, leaf; R, root; and S, stem

Name	Formula	Molecular	RT	Class	Measured				R vs F		R vs L		R vs S	
		Weight	[min]		R	F	L	S	VI*P* value	*P*-value	VI*P* value	*P*-value	VI*P* value	*P*-value
Saikosaponin A	C_42_H_68_O_13_	780.98	16.13	triterpenoids	7.08E+05	1.98E+04	9.28E+03	5.62E+05	-	-	2.80	0.006	2.07	0.027
Saikosaponin B	C_43_H_72_O_14_	813.02	15.40	triterpenoids	7.29E+07	9.19E+03	1.31E+06	4.55E+03	4.76	0.006	1.77	-	5.61	0.004
Saikosaponin D	C_42_H_68_O_13_	780.98	17.07	triterpenoids	1.94E+08	2.38E+04	6.20E+06	5.60E+04	4.36	0.001	2.17	0.004	5.49	0.000
Momordin Ic	C_21_H_32_O_15_	764.94	13.71	triterpenoids	7.18E+03	5.23E+03	4.90E+03	1.45E+04	-	0.008	-	-	-	-
Betulonicacid	C_42_H_68_O_13_	454.68	9.85	triterpenic acid	7.57E+04	4.99E+03	5.89E+04	1.41E+05	-	-	-	-	1.27	0.032
Kaurenoic acid	C_23_H_28_O_11_	302.45	6.51	diterpenoid	3.03E+04	5.02E+03	1.03E+05	1.75E+05	-	-	1.00	-	1.27	0.032
Rehmannioside A	C_30_H_46_O_3_	524.47	8.56	monoterpenoids	4.74E+03	2.44E+04	8.28E+04	3.81E+04	1.02	0.026	1.77	0.004	-	0.046
Albiflorin	C_40_H_56_	480.46	10.31	monoterpenoids	3.66E+03	6.30E+03	4.21E+03	7.78E+03	-	-	-	-	-	-
Artemisinin	C_24_H_28_O_4_	282.33	19.22	sesquiterpene	2.64E+05	8.82E+04	5.11E+04	7.85E+04	-	0.000	1.13	0.000	-	0.000
Atractylenolide I	C_15_H_22_O_5_	230.30	13.35	sesquiterpene	6.60E+05	4.01E+04	7.29E+03	2.05E+05	-	-	2.80	0.005	1.28	-

- means none significant (*VIP value* > 1.00 or *P-value* >0.05)

HPLC analysis revealed that the relative abundance of SSs differed among the four organs (Additional file 1: Table S2). The abundance of the SSd and SSa was only found in root.

### Transcriptome analysis of the root, stem, leaf and flower

Through RNA-Seq, a total of 12 samples in the four organs independently yielded numbers of raw RNA-Seq reads ranging from 40,761,460 to 77,506,190 (Table 2). After stringent quality checks and removal of adaptor sequences, clean paired-end reads ranging from 40,243,798 to 76,382,598 were acquired. Then, the expression profiling of genes involved in the saikosaponin biosynthetic pathway was subsequently performed. Between 69.84 and 81.54% of the clean reads were mapped. Finally, a total of 93,486 unigenes were assembled (Additional file 1: Table S3).

**Table 2:** Summary of transcriptome data generated in root, stem, leaf, flower

Sample name	Raw reads	Clean reads	Clean bases(G)	Error rate(%)	Q20(%)	Total mapped
root1	61,923,840	61,278,296	9.19	0.02	98.22	49347822(80.53%)
root2	59,085,774	56,835,612	8.53	0.03	97.71	46164456(81.22%)
root3	50,949,232	50,224,936	7.53	0.02	98.20	39732948(79.11%)
stem1	52,918,430	52,169,174	7.83	0.02	98.19	41240630(79.05%)
stem2	49,945,800	49,481,408	7.42	0.02	98.36	40467226(81.78%)
stem3	49,924,560	49,120,966	7.37	0.02	98.31	39130028(79.66%)
leaf1	40,761,460	40,243,798	6.04	0.02	98.34	32651514(81.13%)
leaf2	53,021,426	52,225,618	7.83	0.02	98.13	42585984(81.54%)
leaf3	52,257,396	51,626,318	7.74	0.02	98.14	41497244(80.38%)
flower1	77,506,190	76,382,598	11.46	0.02	98.15	53983590(70.68%)
flower2	53,204,672	52,616,858	7.89	0.03	98.10	37283804(70.86%)
flower3	56,907,780	56,118,732	8.42	0.02	98.09	39192864(69.84%)
total	658,406,560	648,324,314				

To investigate the functions of the unigene set transcripts, a sequence similarity search was conducted using protein databases including the non-redundant (nr), Swiss-Prot, and Pfam databases using BLASTx (cutoff E-value <1e^− 10^). The results indicated that 89,488 (95.72%), 78,155 (83.60%), and 61,248 (65.52%) transcripts shared significant similarity with protein sequences in the nr, Swiss-Prot, and Pfam databases, respectively. In addition, we used BLAST to search the nucleotide sequence databases, including the nucleotide (nt) database, and 81,779 (87.48%) transcripts were also significantly similar to nucleotide sequences.

**Fig. 3:**
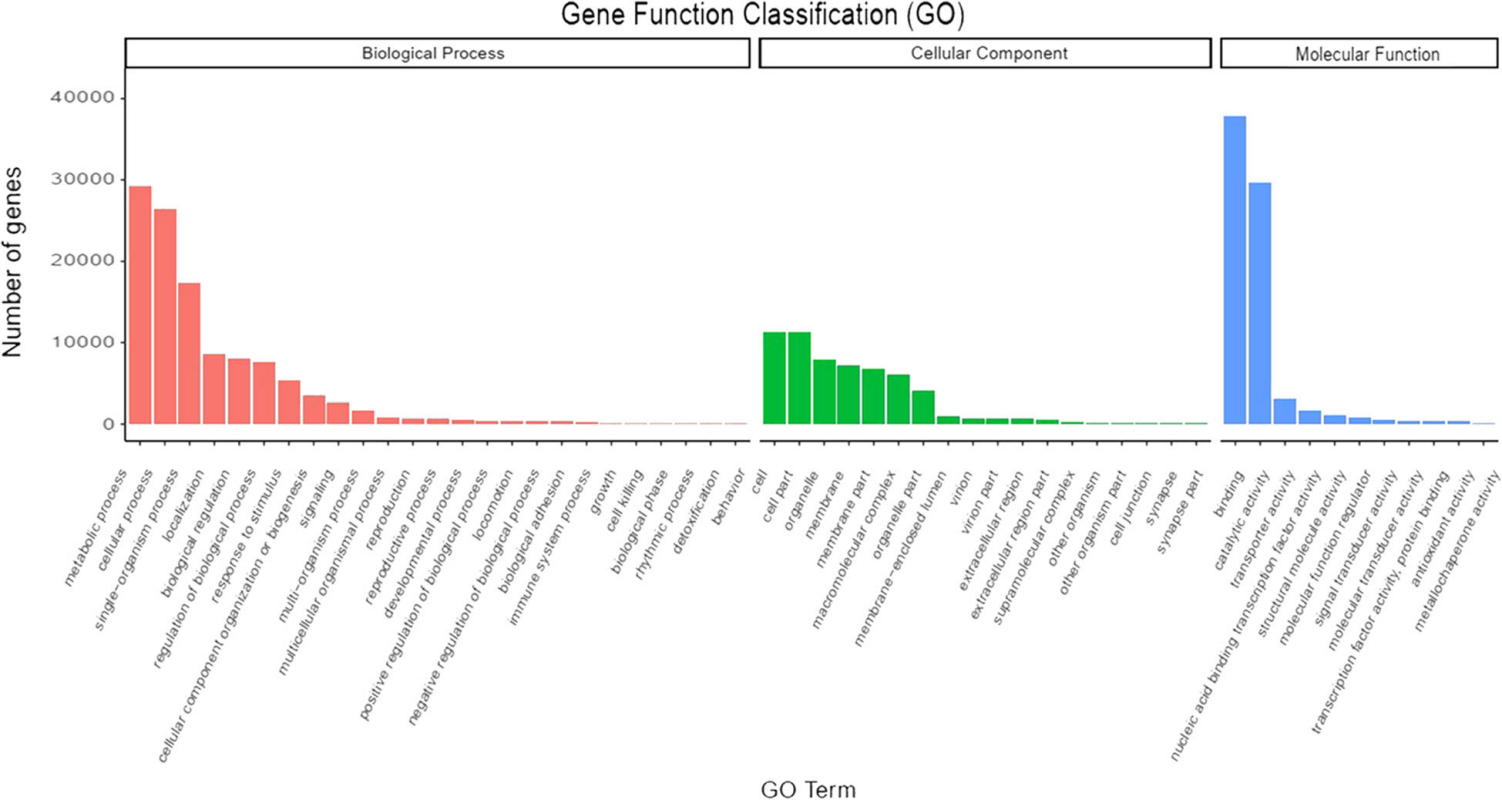
Gene ontology (GO) classification of the unigenes derived from CBC1

Gene Ontology (GO) analysis was performed to classify the functions of the assembled transcripts. Based on the GO analysis, a total of 61,248 (65.52%) unigenes were assigned to 2137 GO terms. The top GO terms of all the transcripts were categorized into 54 functional groups in three main categories, namely, the biological process, molecular function, and cellular component categories. As shown in Fig. 3, in the biological process category, the most highly encoded proteins were those associated with the metabolic process term (GO:0008152, 29,115 unigenes, 47.54%), followed by those associated with the cellular process (GO:0009987, 43.00%) and single-organism process (GO:0044699, 28.21%) terms, indicating that the plants were undergoing extensive metabolic activity. In the molecular function category, binding (GO:0005488, 38,826 unigenes, 61.76%), catalytic activity (GO:0003824, 48.41%) and transporter activity (GO:0005215, 4.97%) were well-represented terms. In the cellular component category, the genes were mostly enriched for the cell (GO:0005623, 11,179 unigenes, 18.25%) and cell part (GO:0043226, 18.25%) terms.

### Comparison of saikosaponin synthesis pathways among the root, stem, leaf, and flower

In order to better understand saikosaponin synthesis pathways, all the unigenes were analyzed using the Kyoto Encyclopedia of Genes and Genomes (KEGG) pathway database. KEGG database contains records of triterpenoid pathways and molecular interactions in organisms cells. In total, 241 and 114 unigenes were annotated in the terpenoid backbone biosynthesis (map 00900) and sesquiterpenoid and triterpenoid biosynthesis (map 00909) pathways, respectively.

**Fig. 4:**
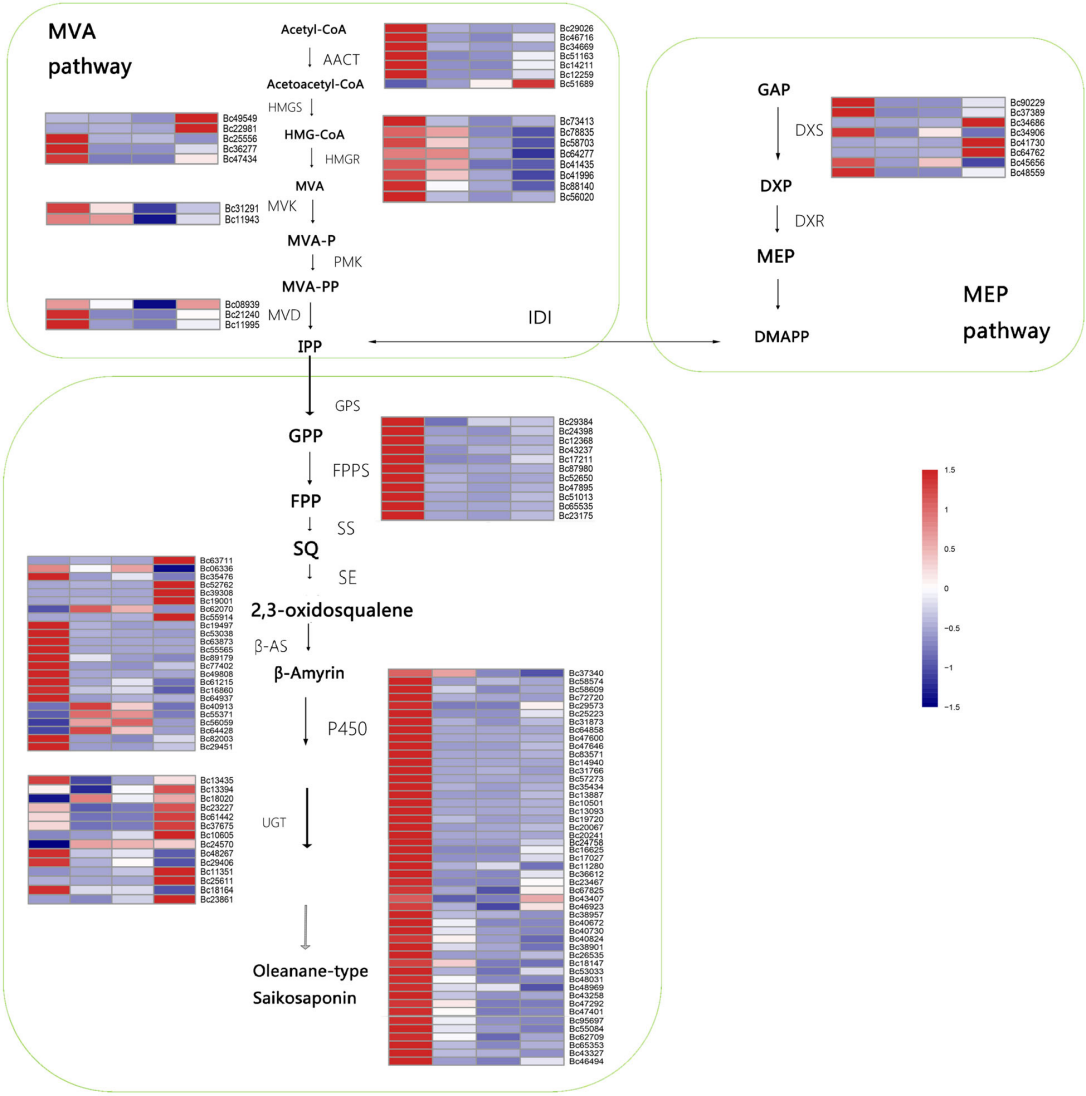
Differential expression of genes among the root, stem, leaf and flower in the saikosaponin biosynthetic pathway. AACT, acetoacetyl CoA transferase; HMGS, HMG-CoA synthase; HMGR, HMG-CoA reductase; MVK, mevalonate kinase; PMK, phosphomevalonate kinase; MVD, mevalonate diphosphate decarboxylase; DXS, 1-deoxy- D -xylulose-5-phosphate synthase; DXR, 1-deoxy- D -xylulose 5-phosphate reductoisomerase; IDI, isopentenyl-diphosphate delta-isomerase; GPS. geranifolium diphosphate synthetase; FPPS, farnesyl diphosphate synthase; SS, squalene synthase; SE, squalene epoxidase; β-AS, β-amyrin synthase; P450, cytochromes P450; UGT, uridine diphosphate glycosyltransferases

To seek for genes involved in the differential catabolism of saikosaponin in four organs, the abundance and expression patterns of all gene transcripts were analyzed based on their fragments per kilobase of exon per million fragments (FPKM) transcriptome data. Differentially expressed genes (DEGs) in the MVA pathway were found in transcriptome through individual assembling data and were divided into five families: the AACT family (7 unigenes), the HMGS family (5 unigenes), the HMGR family (8 unigenes), the MVK family (2 unigenes), and the MVD family (3 unigenes). For the DEGs involved in the MEP pathway, only one gene family, the DXS family (8 unigenes), was identified. The triterpene biosynthesis-related DEGs included SS (11 unigenes), β-AS (24 unigenes), P450s (49 unigenes) and UGTs (14 unigenes), which catalyze the conversion of oleanane-type SSs (Fig. 4; Additional file 1: Table S4).

### Correlations between unigenes and metabolites

In order to understand the regulatory network for the differential distribution of SSs among roots, stems, leaves and flowers, the relative abundance of SSa, SSb and SSd was determined in the metabolite study, and Pearson correlation analysis was performed with 131 DEGs that were categorized into saikosaponin synthetic pathways. The correlation analysis results showed that 96 unigenes had strong correlation coefficient values (*P* < 0.05) with 3 metabolites. Based on the results, networks of interactions among the metabolites and unigenes were organized for the roots, stems, leaves and flowers.

**Fig. 5:**
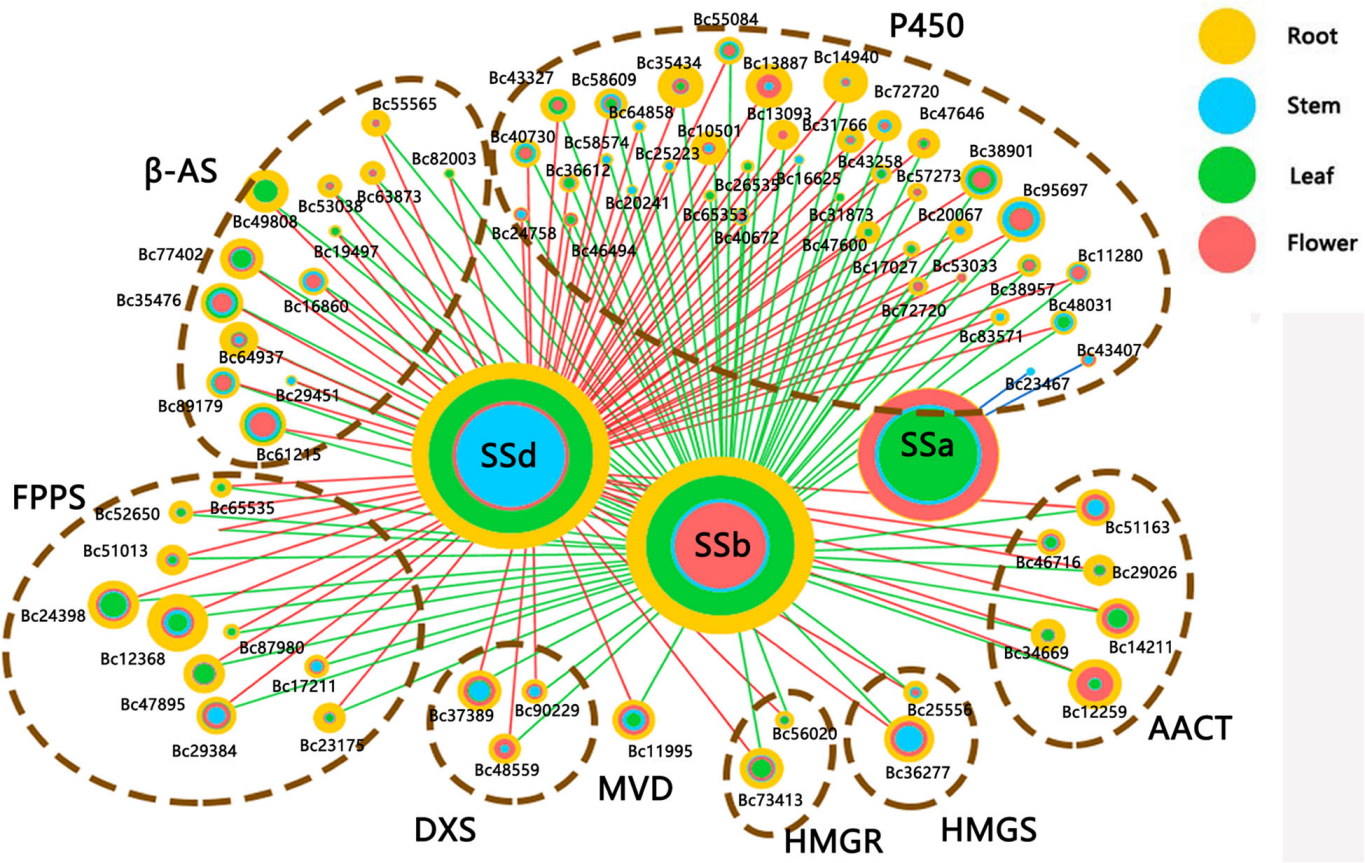
Connection network between regulatory genes and saikosaponins. AACT, acetoacetyl CoA transferase; HMGS, HMG-CoA synthase; HMGR, HMG-CoA reductase; MVD, mevalonate diphosphate decarboxylase; DXS, 1-deoxy- D -xylulose-5-phosphate synthase; FPPS, farnesyl diphosphate synthase; β-AS, β-amyrin synthase; P450, cytochromes P450

The relative abundance levels of SSb and SSd were greater in roots than in other organs and were highly correlated with the unigenes in the AACT, FPPS, HMGR, β-AS, MVD and P450s clusters (Fig. 5). On the other hand, SSa was more predominant in flowers than in other organs and was shown to be highly connected with the unigenes in the P450s cluster.

### Real-time polymerase chain reaction (PCR) validation

**Fig. 6:**
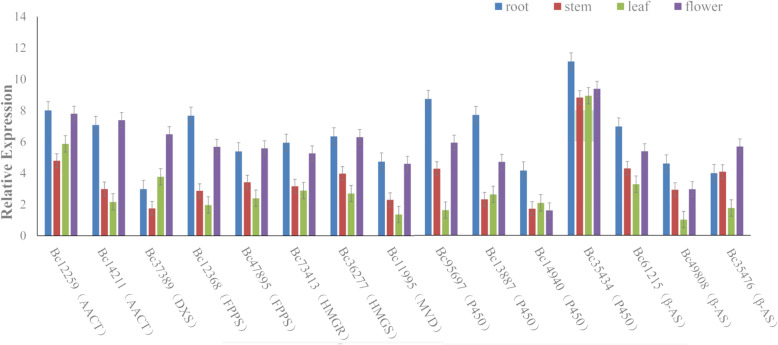
Saikosaponin synthesis in the flower, root, leaf, and stem of B. chinense. Error bars for three technical replicates represent the standard error of the mean (the expression of *Bc52212* in root as reference ΔCt). AACT, acetoacetyl CoA transferase; DXS, 1-deoxy-D-xylulose-5-phosphate synthase; FPPS, farnesyl diphosphate synthase; HMGR, HMG-CoA reductase; HMGS, HMG-CoA synthase; MVD, mevalonate diphosphate decarboxylase; P450, cytochrome P450; β-AS, β-amyrin synthase

Real-time PCR was used to experimentally verify the relative expression of putative unigenes involved in the biosynthesis of saikosaponin of *B. chinense*. Fifteen unigenes including the genes putatively encoding enzymes involved in the gene families of AACT, FPPS, HMGR, HMGS, MVD, P450 and β-AS were tested among different tissues of the plant with three replicates (Fig. 6). The real-time PCR analysis suggested that 11 of fifteen unigenes displayed a higher transcript pattern in root, as compared with other organs; whereas only four genes, *Bc14211* (encoding AACT), *Bc37389* (encoding DXS), *Bc47895* (encoding FPPS) and *Bc49808* (encoding β-AS) were noticed a higher level in flowers. High expression level of these unigenes in the roots of the plant indicated that SSs are highly produced in the roots. These results were in keep with RNA-seq information analysis.

### Subcellular localization of P450s in *N. benthamiana* cells

**Fig. 7:**
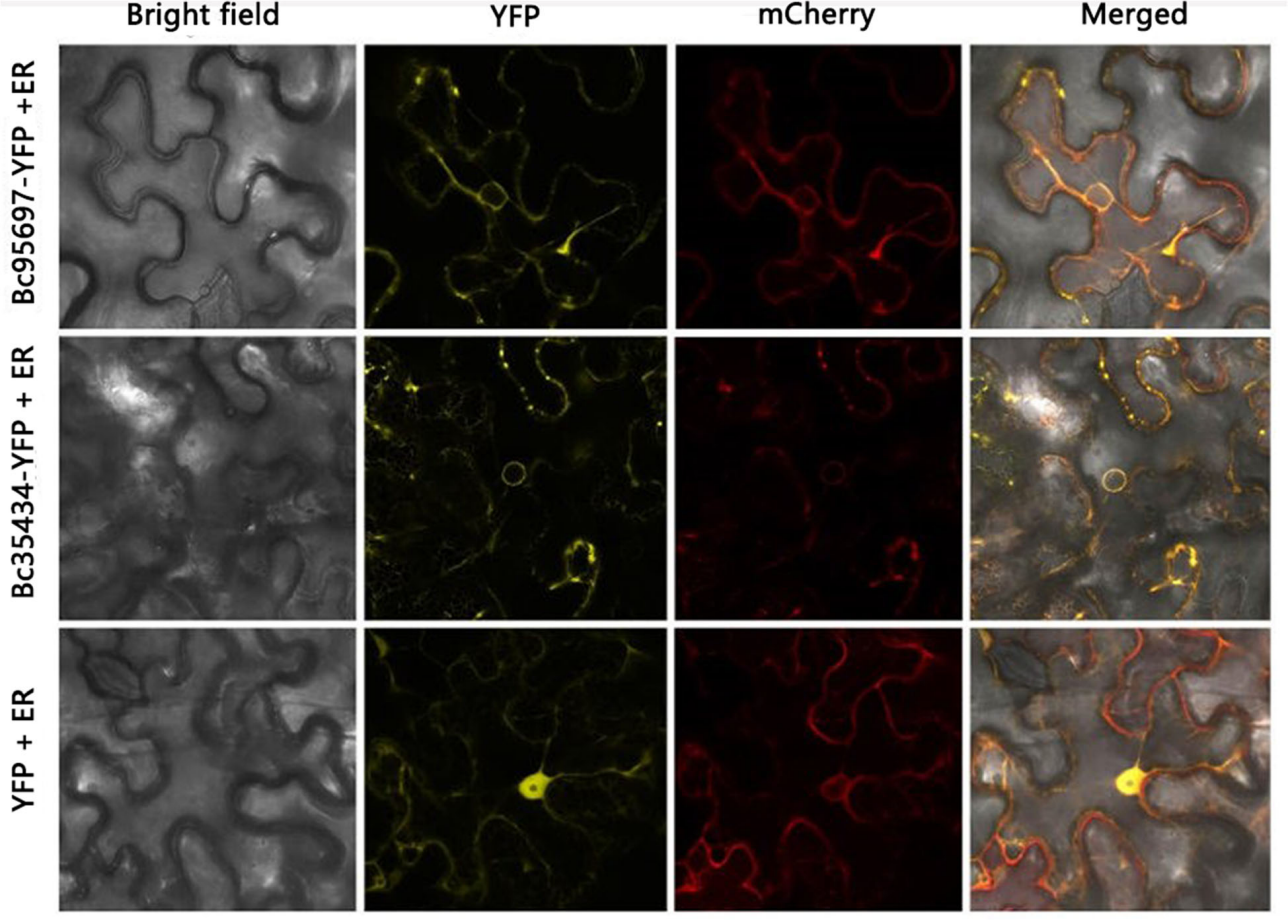
Nuclear localization of Bc95697- and Bc35434-YFP fusion proteins

*Bc95697* and *Bc35434* are both genes encoding P450s. In order to understand their function, the subcellular localization was analyzed. Confocal microscope observation showed that controls expressing a 35S-yellow fluorescent protein (YFP) construct showed fluorescence labeling in endoplasmic reticulum and nucleoplasm. The fluorescence signal of Bc95697-YFP and Bc35434-YFP fusion protein in tobacco epidermal cells are obviously located in the endoplasmic reticulum (Fig. 7), indicating that Bc95697 and Bc35434 are active proteins in the endoplasmic reticulum.

### The high content of two saikosaponins in four genetic types accompanied with the tissue-specific expression of *Bc95697* and *Bc35434* genes

**Fig. 8:**
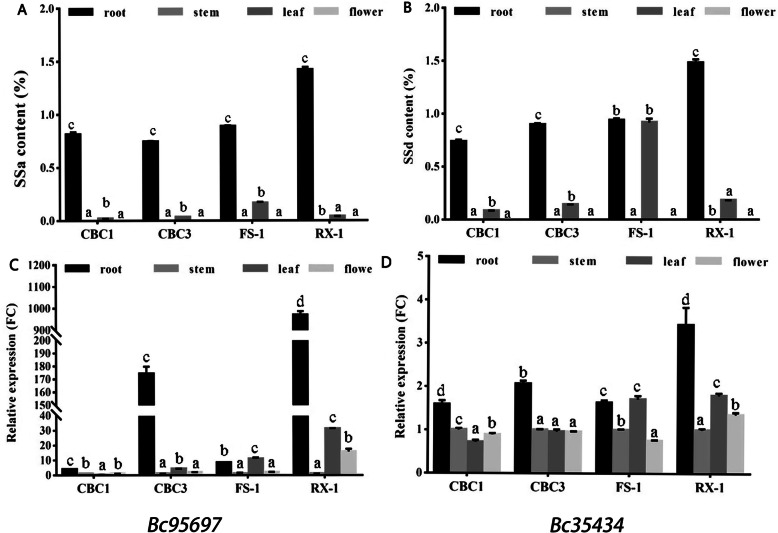
The tissue differences of saikosaponin content and two P450s unigenes expression patterns among four genotypes *Bupleurum.* Tissue specific synthesis of SSa and SSd in four genotypes *Bupleurum*. **A** and **B**. The expression pattern of *Bc95697* and *Bc35434* (**C** and **D**). Different letters indicate significant difference (*P* < 0.01), *n* = 5, Duncan test was used for analysis. SSa, Saikosaponin A; SSd, Saikosaponin D

To explore the roles of *Bc95697* and *Bc35434*. Two genes from CBC1, CBC3, FS-1 and RX-1 were analyzed by real-time PCR analysis. We found the expression of *Bc95697* and *Bc35434* at different genetic type of *Bupleurum* has tissue-specific expression, and the expression in root was obviously higher than in other organs (Fig. 8). In line with the higher expression of the genes, liquid chromatography-mass spectrometry (LC-MS) analysis suggested that the abundance of SSa and SSd showed a significant higher (*P* < 0.01) in root.

## Discussion

We characterized 10 terpenoids in four organs, four of which were triterpenes. Oleanane-type SSs (SSa, SSd and SSb) formed the major group of triterpenes in the root, leaf, and flower of the plant, which is consistent with a previous report that SSa and SSd are the most abundant triterpene saponins found in *Bupleurum* species [12]. SSs with variations in component abundance and total saponin content among different plants are easily affected by the environment [13]. Previous methods such as thin-layer chromatography (TLC) and HPLC, were limited to detect a few active components with relative high abundance, such as SSa, SSc and SSd [13]. The continual refinement and increasing scope and scale of plant metabolite profiling methods have led to the evolution of modern plant metabolomics, which has matured as a valuable tool for advancing our understanding of plant biology and physiology [14]. This detection technique has been successfully applied for the determination of a variety of ginsenosides in *Panax ginseng* [11]. In the present study, only SSa and SSd were detected in roots by HPLC; we deduced the levels of three SSs types in four organs through a metabolomics method. Therefore, this method could help establish a platform for the detection of secondary metabolites in *B. chinense*.

To identify the key genes associated with the differences in saikosaponin levels among organs, we analyzed the expression levels of the 131 unigenes based on FPKM values and compared their expression patterns. The results indicated that four proteins encoding acetoacetyl CoA transferase, HMG-CoA synthase, HMG-CoA reductase and mevalonate diphosphate decarboxylase were significantly correlated (*P*-value < 0.05) with SSd and SSb. In the MVA pathways, AACT is the first enzyme, while HMGR is the first rate-limiting enzyme [15]; therefore, these enzymes might perform important functions in the accumulation of triterpenes. We found that the expression of unigenes *Bc12259* (encoding AACT) and *Bc73413* (encoding HMGR) in roots was higher than that in other organs. In contrast, except DXS family genes, the unigenes involved in the MEP pathway have no differential expression. Three unigenes (*Bc37389*, *Bc90229*, *Bc48559*) had significant correlations with SSd and SSb. These results are consistent with the findings of other studies showing that the MVA pathway generally supplies precursors for the production of sesquiterpenes and triterpenes and that the MEP pathway generally supplies precursors for the biosynthesis of diterpenoids and carotenoids [16].

With regard to triterpene biosynthetic pathway genes, eleven FPPS unigenes were significantly correlated with SSb and SSd. Among the genes in this family, *Bc12368* had the highest expression of roots. In another study on the *Bupleurum* genus, FPPS expression levels and saikosaponin production increased rapidly when the hairy root of *B. falcatum* was cultured in 3XRCM [17]. Various studies have explored the functions of FPPS in plant sources [18–20] and confirmed that the synthase catalyzes the reaction of two molecules of IPP with dimethylallyl diphosphate (DMAPP), resulting in geranyl diphosphate (GPP). Besides, all *β-AS* unigenes were highly expressed in the root. These results are in accordance with the root of *B. chinense* being the most active organ for terpene biosynthesis. The unigene *Bc61215* (encoding β-AS) was significantly correlated with SSd and SSb. Recently, a genome-wide analysis of *Platycodon grandiflorus* revealed that the β-AS gene family played an important role in platycoside biosynthesis, and it had species-specific amplification [21]. In *B. kaoi*, exogenous application of MeJA increases the expression of β-AS (by 2-fold) and glycosyltransferase (by 97-fold), which very likely contributes to increases in SSs [22]. β-AS has thus been defined as an important branch point between primary and secondary metabolism, and might play a regulatory role in the control of triterpenoid saponin biosynthesis. The cDNAs of a few β-AS have been cloned, including two different isoforms of β-AS from *B. chinense* and one from *B. kaoi* [7, 23].

The hydroxylation and oxidative modification are key factors for immense structural diversity of triterpenoids in triterpenoid biosynthesis, and P450s, which mediate oxidations, is the key enzyme. *P450s* of different P450 gene families, which represent five (CYP 51, 71, 72, 85 and 86) P450 clans, are identified as participating in the synthesis of triterpenoid secondary metabolites [24]. Phylogenetic analysis of more than 400 *CYP716s* from more than 200 plant and its heterologous expressions in engineering yeasts showed that subgroup A, E, Y, S and C of *CYP716* family played an important role in triterpenoid diversity [25]. CYP716Y1, a P450 from *B. falcatum* was found to catalyzes the C-16α hydroxylation of amyrin [2]. In our study, *Bc95697* and *Bc35434* (encoding P450s) were significantly correlated with SSb and SSd. *Bc95697* belongs to subclass Y, which is highly homologous to *CYP716Y1*, while *Bc35434* belongs to subclass A. Through real-time PCR analysis, the two single genes were highly expressed in plant roots. In addition, the expression levels of these two unigenes in different tissues with multiple genotypes is also highly correlated with the synthesis of saikosaponin. The subcellular localizations of Bc95697 and Bc35434 were localized in the endoplasmic reticulum. Therefore, these two unknown candidate unigenes may be involved in the saikosaponin triterpene biosynthetic pathway.

The materials in the current study were harvested at the adult stage, and four parts were analyzed at the same time: the root, stem, leaf and flower. Compared with the results of studies conducted on plants in the seedling stage, our results can better reflect the synthesis of SSs. This integrated comparison between metabolite contents and the expression of unigenes involved in its biosynthesis demonstrates that biosynthesis of terpenes in CBC1 is highly controlled by the transcription regulation of the above genes. Furthermore, all the identified rate-limiting enzymes are expected to be useful for various breeding studies on *B. chinense* and possibly other plant species.

## Conclusions

In the current study, we investigated the diversity of the saikosaponin triterpene biosynthetic pathway in the roots, stems, leaves and flowers of *B. chinense* by integrated transcriptomic and metabolomic analysis. Genes from the AACT, FPPS, HMGR and β-AS families were found to play vital roles in regulating saikosaponin biosynthesis, and were related to variations in saikosaponin levels. *Bc12259* (encoding AACT), *Bc73413* (encoding HMGR), *Bc12368* (encoding FPPS), *Bc61215* (encoding β-AS), *Bc95697* and *Bc35434* (encoding P450s), which are responsible for the metabolic diversity of these *Bupleurum* organs, are good candidates to be utilized for the genetic improvement of this important medicinal plant.

## Methods

### Plant materials

The varieties of *B. chinense* genotypes of CBC1, CBC3, FS-1 and RX-1 were applied in current experiment. All of them were bred by Professor Jianhe Wei at the Institute of Medicinal Plant Development (IMPLAD), Chinese Academy of Medical Sciences & Peking Union Medical College (Beijing, China), and Dr. Ma Yu from Southwest University of Science and Technology (Mianyang, Sichuan, China). CBC1 was the commercial variety of Chuanbeichai No. 1, which originated from northern region of China [26]. CBC3, FS-1, and RX-1 were originated from high rainfall areas of Sichuan province and selected as sources of high sclerotinose resistance. CBC3 was collected from a forest (800 m) in Wangcang in October 2016. Fs-1 was collected from a medicinal plant farm (1100 m) in Qingchuan in November 2013. RX-1 was collected from a medicinal plant farm (600 m) in Rongxian in October 2013. The species of CBC3 was identified by Professor Zechun (Chengdu Institute of Biology, Chinese Academy of sciences, Chengdu, Sichuan, China). FS-1 and RX-1 were identified by Professor Jianhe Wei. The voucher specimens of CBC1 (No.0320120003), CBC3 (No.0320160171), FS-1 (No.0320130129) and RX-1 (No.0320130137) were deposited in the herbarium of School of life science and engineering, Southwest University of Science and Technology, Mianyang, Sichuan, China. All genotypes were planted in a 150 cm × 400 cm plot at 25 cm intervals in the greenhouse (14 h light/10 h dark at 25 °C) in Mianyang (31°32′N and 104°42′W), Sichuan Province, China. The roots, stems, leaves, and flowers of the plants were collected at the flowering stage in June 2018 and 2020; three biological replications were assessed for each organ. The samples were frozen immediately in liquid nitrogen and kept at − 80 °C for long-term use.

### Metabolite extraction

Frozen tissues of CBC1 (100 mg) were individually ground with liquid nitrogen, and the ground tissues were resuspended in 500 μL of prechilled 80% methanol (Thermo, United States) and 0.1% formic acid (Thermo, United States) for vortexing. The samples were incubated on ice for 5 min and then centrifuged at 15000 rpm in a refrigerated centrifuge (ST16R, Thermo, United States) at 4 °C for 10 min. Some of the supernatant was diluted to obtain a solution containing 53% methanol (Thermo, United States) with LC-MS grade water. The samples were subsequently transferred to fresh Eppendorf tubes (500 μL) and then centrifuged at 15000×*g* and 4 °C for 20 min. Finally, the supernatants were injected into a HPLC-MS/MS system for analysis. In addition, equal volumes of the different experimental samples were taken and blended as quality control (QC) samples, which were used to evaluate the stability of the results of the whole experimental process and the correlation analysis.

### HPLC-MS/MS analysis

HPLC-MS/MS analyses of CBC1 were performed using an ExionLC™ AD system (SCIEX, United States) coupled with a QTRAP® 6500+ mass spectrometer (SCIEX, United States) [27]. The samples were injected onto a BEH C8 column (2.1 mm × 100 mm, 1.9 μm, Waters, United States) using a 30 min linear gradient at a flow rate of 0.35 mL/min in the positive (ESI+) polarity mode. The eluents were eluent A (0.1% formic acid/water) and eluent B (0.1% formic acid/acetonitrile). The solvent gradient was set as follows: mobile phase B was increased linearly from 5% at 1 min to 100% at 24 min and then held at 100% until 28.0 min. Finally, solvent B was decreased to 5% at 28.1 min and held at 5% until 30 min. The samples were injected onto an HSS T3 Column (2.1 mm × 100 mm, Waters, United States) using a 25 min linear gradient at a flow rate of 0.35 mL/min for the negative (ESI–) polarity mode. The eluent was the same as that for the ESI+ mode. The solvent gradient was set as follows: mobile phase B was increased linearly from 2% at 1 min to 100% at 18 min and then held at 100% until 22.0 min. Finally, solvent B was decreased to 5% at 22.1 min and held at 5% until 25 min.

Mass data acquisition was performed in both ESI+ and ESI– modes using the following parameters: ion spray voltages of 5500 V in ESI+ mode and − 4500 V in ESI– mode, an ion source gas 1 pressure of 55 psi, an ion source gas 2 pressure of 55 psi, a curtain gas pressure of 30 psi, and a turbo spray temperature of 600 °C. The data files generated by HPLC-MS/MS were processed using SCIEX OS Version 1.4 to integrate and correct the peaks. The main parameters were set as follows: minimum peak height, 500; signal/noise ratio, 10; Gaussian smooth width, 3. The area of each peak represented the relative content of the corresponding substance.

### RNA isolation and transcriptome sequencing using the Illumina platform

Total RNA was extracted from the four organs of CBC1 using TRIzol (Invitrogen™, Life Technologies, Carlsbad, United States) according to the manufacturer’s protocol. The quality and quantity of the extracted RNA were assessed using an Agilent Bioanalyzer 2000 (Agilent Technologies, Palo Alto, United States). An RNA-Seq library with a 150-bp insertion size was then constructed and sequenced on the Illumina sequencing platform (Novogene, Beijing, China).

### Extraction of saikosaponins and HPLC analysis

For each organ of four genotypes, three biological replications were independently analyzed. In total, 12 samples were randomly analyzed to reduce analysis bias. The SSa and SSd content was determined using a Waters HPLC system (Waters 1525 Binary HPLC Pump, United States) and an Zorbax Extend-C18 column (250 mm × 4.6 mm, 5 μm, Agilent, United States). The reference standards of SSa and SSd were purchased from the National Institutes for Food and Drug Control, Beijing, China. The methods and conditions for determination have been reported previously [28].

### Multivariate statistical analysis

The metabolites were annotated with the KEGG database (https://www.kegg.jp/), the HMDB (http://www.hmdb.ca/) and the LIPID MAPS database (http://www.lipidmaps.org/). The intensities of the mass peaks for each sample were sum-normalized and Pareto-scaled using metaX software [29]. PCA and partial least squares-discrimination analysis (PLS-DA) with data from 12 samples (four organs × three biological replicates) were performed to observe differences in metabolic composition among the four plant organs. The variable importance in the projection (VIP) values of all metabolites from the PLS-DA were extracted using the first component [30]. We selected metabolites satisfying the following criteria as potential markers: (i) high confidence (VIP value> 1.0) in discriminations between root and stem, between root and leaf, and between root and flower; (ii) mean intensities in one plant organ different from those in another organ (*P*-value < 0.05); and (iii) a minimum of a 2-fold change [31]. The *P*-value was calculated using an independent two-sample t-test.

### Transcript profiles and annotation

The sequence data were processed using SMRTlink 5.1 software. Additional nucleotide errors in consensus reads were corrected using the Illumina RNA-Seq, data with the software LoRDEC [32]. Any redundancies in the corrected consensus reads were removed by CD-HIT to obtain the final transcripts for the subsequent analysis [33]. The mapped clean reads for each gene were counted, and the numbers were normalized using the DESeq2 package in R (3.6.2) [34]. Genes showing significantly different expression among the four organs were identified by fold change and binomial tests using the DESeq2 (log2 > 0 & *P*-value < 0.05). The expression levels of genes were determined based on the FPKM values from RNA-Seq data for roots, stems, leaves and flowers using RSEM [35]. The mean FPKM values of the genes were converted to log2 values, which were used for visualization using the heatmap package in R (3.6.2).

Annotation of the transcriptome was carried out using a BLAST search of nt sequences with an E-value cutoff of 1e^− 10^ and a BLASTx search against the GenBank nr protein [36], Swiss-Prot [37], KEGG [38], KOG [39], Gene Ontology [40], and Pfam (http://pfam.xfam.org/) databases with an E- value cutoff of 1e-10. For each sequence, the top hit was extracted and used for further processing. GO and KEGG pathway functional enrichment analyses were performed by the GOseq R package (3.6.2) and KOBAS software, respectively [41, 42].

### Integrative analysis of the metabolome and transcriptome

Pearson correlation coefficients were calculated for metabolome and transcriptome data integration. For this, the mean content of SSs (SSa, SSb, and SSd) in each organ from the metabolome data and the mean value of the DEGs in each organ from the transcriptome data were used for the correlation analysis. Correlations corresponding to a coefficient with R^2^ > 0.9 (*P*-value < 0.05) were selected (Additional file 1: Table S5). Cytoscape (version 3.6.1) [43] was used to visualize the final interaction network.

### Real-time PCR analysis

Fifteen genes potentially involved in the saikosaponin synthesis of *B. chinense* were selected to perform expression pattern analysis. Total RNA was extracted by TRIzol (Invitrogen™, Life Technologies, Carlsbad, United States) from independent biological samples collected at the same stage as the ones used for RNA-Seq analysis and using as described above. The cDNAs were synthesized by the TransScript All-in-One First-Strand cDNA Synthesis SuperMix kit (TransStart, Beijing, China) using specific primer pairs [44] (Additional file 1: Table S6). Real-time PCR was performed using CFX96 Touch Real-Time PCR Detection System (Bio-Rad, Hercules, CA, USA) with Green qPCR SuperMix UDG kit (TransStart, Beijing, China). The PCR reactions were conducted as follows: 30 s at 95 °C for denaturation, 5 s at 94 °C, 15 s at 60 °C for annealing, and 10 s at 72 °C for extension. The relative abundance of transcripts was calculated using the 2 ^–∆∆Ct^ formula [45]. In the assays, the β-action gene *BcADF7* was used as the housekeeping gene for normalization. The experiments were performed for three technical replications.

### Subcellular localization

To further understand the biological roles of P450s enzymes, we conducted the intracellular localization of P450s expressed with YFP fusions transiently in *Nicotiana benthamiana* leaves by confocal microscopy. The fusion expression vector of *Bc95697* and *Bc35434* genes were constructed using specific primer pairs (Additional file 1: Table S6). The two DNA segments were inserted into the pC131-YFP vector to generate Bc95697-YFP and Bc35434-YFP fusion proteins. The two recombinant vectors were transferred into *Agrobacterium tumefaciens* GV3101 strains and suspended in the mixture solution with 10 mM MgCl_2_, 10 mM MES, and 100 μM acetosyringone (Sigma-Aldrich), pH 5.6. After incubation for 3 h at room temperature, the strain was transfused into leaves of *N. benthamiana* plants. Fluorescence images of the YFP and mCherry signals were visualized using a laser scanning confocal microscope (Leica TCS SMD FCS, Germany) after 3 days. The excitation and wave-length emissions were 514 and 530–580 nm for YFP, 587 nm and 610–650 nm for mCherry, respectively.

## Supplementary Information

**Additional file 1: Table S1.** A list of 600 metabolites identified in four organs. **Table S2.** Mean content of Saikosaponin A (SSa) and Saikosaponin D (SSd) among the root, stem, leaf, flower. **Table S3.** Summary of unigene ID and sequence of *B.Chinese*. **Table S4.** FPKM values of unigenes involved in the saikosaponin synthesis pathways among the root, stem, leaf and flower*.* **Table S5.** Interaction value between metabolite and gene. **Table S6.** Primer list used in real-time PCR analysis.

## Data Availability

The raw sequence data generated in this study have been deposited in the Short Read Archive (SRA) of the NCBI under accession number PRJNA728560 (https://dataview.ncbi.nlm.nih.gov/object/PRJNA728560).

## Abbreviations

IPP: Isopentenyl diphosphate
DMAPP: Dimethylallyl diphosphate
MVA: Mevalonate
MEP: Methylerythritol phosphate
AACT: Acetoacetyl CoA transferase
HMGS: HMG-CoA synthase
HMGR: HMG-CoA reductase
MVK: Mevalonate kinase
PMK: Phosphomevalonate kinase
MVD: Mevalonate diphosphate decarboxylase
DXS: 1-deoxy- D -xylulose-5-phosphate synthase
DXR: 1-deoxy- D -xylulose 5-phosphate reductoisomerase
IDI: Isopentenyl-diphosphate delta-isomerase
GPS: Geranyl pyrophosphate synthetase
FPPS: Farnesyl diphosphate synthase
SS: Squalene synthetase
SE: Squalene epoxidase
β-AS: β-amyrin synthase
P450s: Cytochrome P450 enzymes
UGTs: Uridine diphosphate glycosyltransferases
SSs: Saikosaponins
SSa: Saikosaponin A
SSd: Saikosaponin D
SSb: Saikosaponin B
nr: Non-redundant
nt: Nucleotide
GO: Gene ontology
KEGG: Kyoto Encyclopedia of Genes and Genomes
DEGs: Differentially expressed genes
HPLC-MS/MS: High-performance liquid chromatography-tandem mass spectrometry
QC: Quality control
RNA-seq: RNA sequencing
PCA: Principal component analysis
PCR: Polymerase chain reaction
YFP: Yellow fluorescent protein
LC-MS: Liquid chromatography-mass spectrometry
PLS-DA: Partial least squares-discrimination analysis
VIP: Variable importance in the projection
